# A Group Motivational Interviewing intervention in schools to reduce sugar sweetened beverages intake among young people (DISS): a feasibility study

**DOI:** 10.3389/fpubh.2025.1524574

**Published:** 2025-07-08

**Authors:** Huda Yusuf, Maria Josefina Valenzuela

**Affiliations:** ^1^Institute of Dentistry, Queen Mary University of London, London, United Kingdom; ^2^Institute of Dentistry, Queen Mary University of London, London, United Kingdom

**Keywords:** child obesity, sugar, feasibility study, GMI, prevention

## Abstract

**Introduction:**

Child obesity is a global public health issue affecting high, middle and low-income countries. The causes of obesity are complex and influenced by environmental and behavioural factors including diet and sedentary lifestyles. Young people consume sugar sweetened drinks (SSB) regularly, providing the largest single source of free sugars in the UK. This paper presents the protocol for a cluster feasibility study that aims to assess feasibility of a school obesity intervention delivered by teachers to reduce SSB consumption among 12–13-year-olds in secondary schools to prevent weight gain by using Group Motivational Interviewing.

**Methods:**

An exploratory mixed-methods design will be used. The aim is to recruit six schools (two control and four intervention) in East London and around 720 pupils. The intervention consists of three components. First, teachers will be upskilled in group motivational interviewing to deliver a series of activities to young people during school time. Second, a mobile app for young people will be developed to support the classroom activities. Lastly, a booklet with healthy lifestyle resources will be developed and sent home for parents. The outcome measures for the feasibility study will include the recruitment and retention rates, barriers and facilitators to intervention implementation, and acceptability among teachers and young people. Additionally, anthropometric, dietary, and lifestyle behaviour measures will be collected at baseline and at 6 months to assess acceptability in preparation for a main trial.

**Results:**

The study findings will be submitted for publication in international peer-reviewed journals and shared at international conferences.

**Discussion:**

The results of the study are expected to inform the development of a future trial and to contribute to research on the prevention of weight gain among young people and the reduction of SSB consumption.

## 1 Introduction

Child obesity is a global public health issue affecting high, middle and low-income countries, with varying rates across different regions. In 2022, over 390 million children and young people (5–19-year-olds) were overweight of which 160 million were living with obesity (1). There are significant health inequalities in the rates of childhood obesity with increased risk of non-communicable diseases such as diabetes, cardiovascular and respiratory diseases. Additionally, psychosocial impacts, stigma and discrimination impact on young people. The economic global costs of overweight and obesity are estimated to increase to US$ 3 trillion per year by 2030 (2).

The causes of obesity are complex and influenced by environmental and behavioural factors including diet and physical activity and sedentary lifestyles. One of the main drivers of obesity in children is due to increased intake of sugar sweetened drinks (SSBs), which is also associated with increased risks of dental caries (3). Free sugars intake has risen by 23% among 3–19-year-olds from 1990 to 2018 (4). The mean intake of SSBs among children and adolescents was 3.6 [standardised serving of 248 g (8 oz)] servings/week (95% UI 3.3–4.0) globally. In England, 70% of young people (11–18-year-olds) consume SSBs regularly, providing the largest single source of free sugars (5).

In East London, the prevalence of overweight and obesity among 10–11-year-olds is higher than the national (36.6%) and London averages (38.8%), putting Tower Hamlets (43.1%) in the top 5 boroughs in London for the highest rates (6). The prevalence of obesity for those living in the most deprived areas is twice as high as those living in the least deprived areas. This gap has increased over time. These inequalities should be tackled at the population level ensuring children and adolescents have opportunities for healthy living.

Obesity can be prevented through public health policies, fiscal measures but also supporting families and children towards healthy lifestyles, lowering energy intake from food and increasing physical activity (2, 7).

NICE guidance and Cochrane Reviews have highlighted the lack of obesity interventions targeting 10–14-year-olds in schools (8). This is an important target group as they are old enough to understand and young enough to be influenced (9). Schools are excellent learning environments for health promotion as most children spend one third of their time there.

A meta-analysis of randomised control trials (RCTs) to reduce SSB intake resulted in a weighted mean difference in Body Mass Index (BMI) of −0.12 between control and intervention groups (10). These reductions can be significant at a population level over time. Although the Health Promoting Schools (HPS) Framework can be used to promote healthy behaviour change, evidence on evaluation of interventions to promote health during adolescence is limited. Using personal, social, health and economic (PSHE) education within the curriculum could enhance pupils' skills in achieving healthy lifestyles. Teachers are ideally positioned to promote health; however, they lack core knowledge and skills due to limited training. Psychological techniques such as Group Motivational Interviewing (GMI) could support teachers in facilitating healthy behaviour change in the classroom (11).

GMI has been used effectively in tackling adolescents' drug and alcohol use (12). However, GMI has not been tested for dietary behaviour change. GMI is ideal for adolescents, because teachers can collaborate with them, allowing peer interaction and participation. GMI elicits young people's own ideas about healthy behaviours, thereby respecting personal autonomy (11). Once teachers have acquired GMI skills they could use it universally for other behaviours.

The aim of this study was to assess the feasibility of reducing SSB consumption among 12–13-year-olds in secondary schools to prevent weight gain by using Group Motivational Interviewing. In this paper we present the methodology and intervention design for an exploratory GMI intervention study aimed at reducing SSB consumption in young people attending secondary schools in East London. This is one of the first feasibility studies exploring the use of GMI in SSB reduction among young people in the UK.

## 2 Methods and analysis

### 2.1 Study design, setting, and population

**Figure 1:**
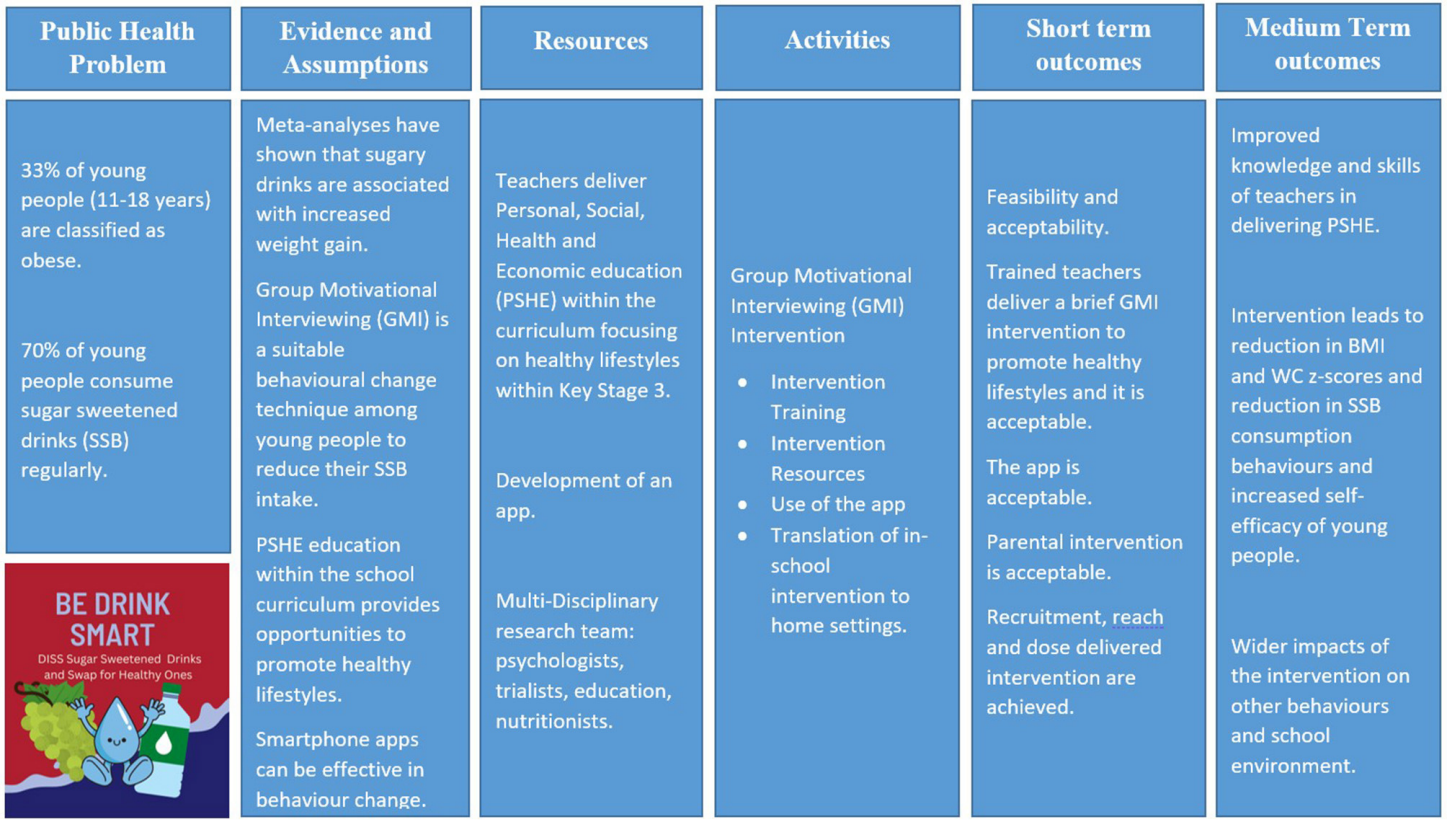
Logic model for the DISS intervention.

The DISS (Diss SSB) study is a cluster feasibility study of an obesity school-to-home intervention delivered by teachers to reduce SSB consumption among 12–13-year-olds in secondary schools across East London. The methodology was guided by the UK Medical Research Council (MRC) guidance on the development and evaluation of complex interventions (13) and advice was sought from the East London Research and Design Service (Figure 1).

The research proposal was informed by consulting with the National Chair of the Personal, social, health and economic education (PSHE) Association's Advisory Council, local teachers, and views of 12–13 year olds who reported that they preferred more collaborative approaches to teaching on PSHE. They wanted teachers to ‘build relationships' with them, which is in line with the ethos of GMI. They reported that mobile apps could be useful in supporting them with behaviour change. The students reviewed the study proposal and also suggested the study acronym “DISS” diss SSB.

#### 2.1.1 Inclusion criteria

- Secondary school teachers to 12–13-year-olds in East London.
- Young people aged 12–13 years old attending state secondary schools (Year 8) in East London. This age group was chosen because:

° High SSB consumption.
° Propensity towards unhealthy behaviours is increased during adolescence, which can shape adult behaviours.
° GMI is effective in adolescents.
° Lessons are delivered in Key stage 3 (12–13 year olds).
° Limited research.

- Parents of Year 8 students.

### 2.2 Sample size

As this is a feasibility trial, it is not necessary to conduct a power calculation. Advice was sought from the Research and Design Service (QMUL) to estimate the sample size. The aim is to recruit six schools (two control and four intervention) in East London and around 720 pupils.

### 2.3 Recruitment of study participants

All state funded secondary schools in East London will be identified using a census sample and invited via email to take part in the study. Emails will be sent to schools, PSHE, and Year 8 leads. Invitation to schools will be also promoted via headteachers newsletters and through public health teams in each of the local authorities. Schools expressing interest will then be contacted and provided with further information. Participating schools will have to provide consent before contacting potential participants as schools are the gatekeepers and have a duty of care to the young person and their staff.

For students and parents, invitation paragraphs will be included in the school's newsletter and posters will be displayed in the school. Age-appropriate information sheets will be sent to, and consent/assent will be sought from teachers, young people and their parents. Only young people with positive assent and consent from their parents/guardians will be able to participate in the data collection process. Data from teachers, young people and parents will be collected in two time points, at baseline and at 6 months follow-up.

### 2.4 Evaluation measures for the feasibility study

It is important to explore key issues at the feasibility testing stage to inform the development of a future trial, including: (a) recruitment and retention rates, (b) barriers and facilitators to intervention implementation, and (c) acceptability among teachers and young people. These factors will be explored using process evaluation frameworks.

#### 2.4.1 Recruitment and retention

Both quantitative and qualitative data will be collected to assess recruitment of participants, including assessing the feasibility of gaining consent from participants. Quantitative data will be collected to determine recruitment, retention and attrition rates.

#### 2.4.2 Dose of GMI delivered

The quantity of the intervention, including the number and duration of sessions, referred to as the dose delivered, will be assessed.

#### 2.4.3 Assessment of acceptability, impacts, barriers and facilitators among study participants

##### 2.4.3.1 Teachers

Two to three focus groups will be conducted with teachers. A topic guide will be developed to assess whether the intervention and measurements tools were acceptable, any barriers or facilitators and any impacts on their knowledge and skills. Purposive sampling will be used based on school locations and control vs. intervention schools.

##### 2.4.3.2 Young people

Two focus groups (6–10 pupils) will be conducted with young people. A topic guide will explore acceptability of measurements, and young person's views about the GMI intervention and its impacts on their knowledge and behaviours.

### 2.5 Piloting primary and secondary health outcomes for the main trial

The feasibility trial is designed to assess the practicality and acceptability of the intervention, while also evaluating the appropriateness of the proposed outcome measures namely body mass index (BMI), waist circumference, and dietary intake assessments to inform a subsequent definitive trial (Table 1).

**Table 1:** Primary and secondary outcomes to assess their acceptability and feasibility in a school setting.

**Outcomes**	**Description**
**Primary outcomes**	
Young person's obesity-related behaviours	Change in young people's mean daily consumption of sugar-sweetened beverages (SSB) at baseline and 6 months
**Secondary outcomes**	
Young person's BMI and Waist circumference	Anthropometric measures (BMI, WC), dietary data, and questionnaires will be collected at baseline and 6 months
Young person's reported physical activity and self-efficacy	Baseline and follow-up questionnaires assessing physical activity, and self-efficacy in changing drinking habits
Teacher's knowledge, skills and confidence	Teachers will complete questionnaires at baseline and follow-up to assess their knowledge, skills, and confidence in teaching health promotion
Parental eating habits and physical activity	Parents will complete self-administered questionnaires at baseline and 6 months

#### 2.5.1 Primary outcomes

##### 2.5.1.1 Young person's obesity-related behaviours

Change in young person's mean daily consumption of SSB, defined as the mean daily total volume (ml) and frequency (number of drinks per day), will be collected through three repeated 24-h dietary recalls at baseline and follow-up using Intake 24. This decision was based on the MRC‘s Diet, Anthropometry and Physical Activity (DAPA) Measurement Toolkit (14).

#### 2.5.2 Secondary outcomes

##### 2.5.2.1 Young person's BMI and waist circumference

Anthropometric measures, dietary measures, and young persons' questionnaires will be collected at baseline and at 6 months to assess their acceptability in preparation for a main trial.

Both BMI and waist circumference (WC) will be measured according to standardised national protocols, using UK reference curves (above 85th centile: overweight and above the 95th centile: obese).

##### 2.5.2.2 Young person's reported physical activity and self-efficacy questionnaires

A brief baseline and follow-up questionnaire will be administered to young people including demographic variables (age, sex, ethnicity, and postcode), intake of SSBs, physical activity, and self-efficacy questionnaire to change drinking habits.

##### 2.5.2.3 Teacher's baseline and follow-up questionnaires

All teachers in the trial will be requested to complete a questionnaire at baseline and follow-up to assess their knowledge, skills and confidence on teaching about health promotion.

##### 2.5.2.4 Parental eating habits and physical activity

Data from parents will be collected using a self-completed questionnaire at baseline and at 6 months follow-up to assess. The questionnaires will collect data about demographics (sex, employment status, and education level), eating habits and physical activity. A folder will be sent to parents with their child with a paper copy of the questionnaire and a QR code to access a digital version of the questions on Microsoft Forms.

### 2.6 Incentives for the study

There will be incentives to schools taking part in the study at a £150 each, incentives for young people will be £5 at baseline and £5 at follow-up. Incentives will also be given to teachers at £30 and to parents £25.

### 2.7 Development of a group motivational interviewing intervention

The intervention was developed by researchers with support from young people, teachers, experts in the field and following consultation with stakeholders from a steering committee.

#### 2.7.1 Patient public involvement

Two advisory councils will be set up in a secondary school in Tower Hamlets to inform the design and co-development of the intervention. One Advisory Council for young people aged 12–13 years and the second with teachers. The aim of these research councils will be to co-develop the intervention and the evaluation framework. The Young People's Advisory Council will allow young people to act as collaborators in the research study by providing their input into the features of the mobile app to support them with behaviour change. The Teachers Advisory Council will inform the co- development of the GMI training and manual.

#### 2.7.2 GMI intervention structure, content and resources

The intervention consists of three components:

- Upskilling of teachers in GMI and inclusion of the intervention into the PSHE curriculum.
- A mobile app for young people to support positive behaviour change.
- Translation of the in-school intervention to a home setting for parents to control SSB intake at home. This will include: a resource pack for parents, and awareness on food labelling.

#### 2.7.3 GMI training for teachers

Teachers will receive two sessions of a training on GMI and healthy living in a school setting. The training consists of two sessions of ~2–3 h each. The first one will be delivered by the research team and will cover health related information. The second session will cover the GMI information which will be delivered by a psychologist with experience in secondary school-based health research, motivational interviewing and behaviour change techniques.

#### 2.7.4 GMI manual

**Figure 2:**
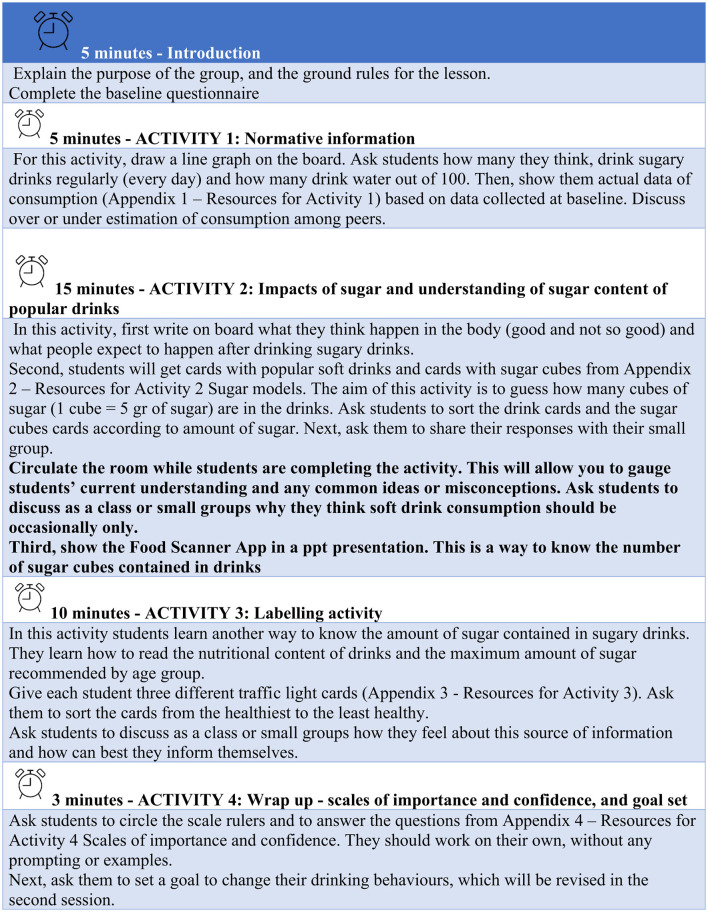
Extract from the activities described in the manual.

A GMI manual will be developed to be used in the delivery of the intervention with support from the teacher's advisory council and a psychologist. The manual will consist of two sections: young people's population profiles in relation to unhealthy weight, the evidence around health interventions and the theory and application of Group Motivational Interviewing principles for behaviour change (Figure 2). The second section will describe in detail the structure of the classroom activities and the activity resources. The manual will be given to teachers after completing the GMI training.

#### 2.7.5 Classroom sessions

The classroom sessions will consist of a series of activities based on group motivational interviewing principles and will be designed to be delivered during PSHE or PE hours in two different days. The first session will be designed to last around 40 min and focused on understanding and identifying the real amount of free sugars in popular SSBs, exploring young people's motivation in changing their drinking habits and encouraging them to set achievable goals for the future. The second session will be designed to last 20 min and focused on increasing the young people's confidence in their ability to change the drinking habits, their commitment to change, as well as exploring their motivations and barriers to change.

#### 2.7.6 Diss dash app

A mobile application, based on Motivational Interviewing principles, will be co-developed with the Young People's Advisory Council to support young people in their behaviour change. The app will contain healthy living resource section, messages to motivate young people to reduce their SSB intake using Motivational Interviewing principles. It is also intended to include a simple game to increase the user's engagement as well as potentially facilitating peer-to-peer interaction. Young people will be able to add their level of confidence and motivation in reducing their intake of SSBs and their own intake goal. The app usage will be monitored on a weekly basis including the frequency of logins, features accessed, duration of use, and engagement in the game.

#### 2.7.7 Take-home lifestyle resources for parents

The third part of the intervention will consist of a booklet aimed at parents. The booklet will contain infographics about healthy living, including healthy eating and physical activity advice from the NHS. The aim of this booklet is to support parents and caregivers to adopt healthier habits at home.

### 2.8 Planned data analysis

Feasibility and acceptability of the intervention will be assessed using an explanatory sequential mixed methods approach (15).

#### 2.8.1 Statistical analysis

The quantitative data analysis will include the report of 95% CIs where appropriate, recruitment rates, attrition rates and questionnaire completion rates. As this is a feasibility study and so not powered for inferential tests of significance, descriptive statistics (eg, as appropriate, mean/median; SD/IQR; frequency, proportion and 95% CI; range) will be used to explore the quantitative data (baseline, follow-up and changes from baseline). Means and 95%CI for mean BMI, mean BMI z-scores, mean WC (cm) and *z*-scores will be reported by control and intervention groups. Means and 95% CI for SSD consumed per day and non-SSBs intake per day by control and intervention groups will be reported.

#### 2.8.2 Qualitative analysis

Verbatim anonymised transcripts of the audio-recorded focus groups will be analysed using a thematic analysis approach (16). This method will involve familiarisation with the data, initial code generation, theme identification, reviewing and refining themes, and defining and naming themes according to the six-phase framework (16).

### 2.9 Ethics and dissemination

Ethics approval has been granted by Queen Mary University of London Ethics Committee to conduct the study (Ref QMERC22.313). The study findings will be shared with the local authorities, and those involves in the research. In addition, the findings will be submitted for publication in international peer-reviewed journals and shared at international conferences.

## 3 Discussion

Young people consume SSBs regularly presenting a growing public health challenge. Although a levy on SSBs has been in place since 2018 in the UK, this needs to be supplemented by additional mid-stream actions including health promoting schools (HPS) and enhancing the skills of young people towards healthy behaviours. However, research in this area has been limited due to the challenges in engaging with secondary schools and young people.

Systematic reviews of HPS-based interventions indicate positive effects on health and educational attainment. Enhancing the knowledge and skills of teachers and utilising Group Motivational Interviewing techniques can be beneficial for teachers and young people in facilitating a collaborative approach and building rapport in achieving positive behaviour change (17).

There is a need to test innovative approaches to weight management interventions targeting young people in school settings, without having to rely on them accessing healthcare or any other settings. Evidence has demonstrated that school-based interventions which target diet and physical activity and incorporating a home component were more effective that those targeting a single behavioural component (18).

It is envisaged that recruitment of secondary schools and young people will be challenging. A recent scoping review on conducting research with young people especially when several gatekeepers such as schools and parents' consent are required (19). There is stigma associated with healthy lifestyles, and it is therefore important to ensure that schools, parents and young people are approached sensitively by ensuring that the research is fun and exciting. To minimise risk of contamination, careful attention will be given to the geographical location of the schools during recruitment, ensuring that intervention and control schools are not in close proximity or part of the same school network. Despite these precautions, there is potential for informal communication between staff or students across schools which cannot be completely avoided.

Schools and specifically teaching staff may have limited resources to be trained and to deliver the intervention. However, advice will be sought from the Teacher's Advisory to ensure minimum disruption to teachers' routines.

The expectation is that an adequate number of schools, teachers and young people will be recruited to evaluate the feasibility and acceptability of the intervention. In order to make recommendations to proceed to a full definitive trial, a feasibility goal of recruiting at least 70% of schools and young people has been established. If the intervention is found to be feasible and acceptable, the next step would be to inform the development of a definitive trial to address the gaps in evidence base in reducing SSB intake among young people and contributing to reductions in the burden of dental disease and obesity.

## Data Availability

The original contributions presented in the study are included in the article/supplementary material, further inquiries can be directed to the corresponding author.
